# Ferroptosis in Breast Cancer: Molecular Insights and Therapeutic Strategies

**DOI:** 10.31083/FBL50950

**Published:** 2026-04-17

**Authors:** Nahid Iftikhar, Suryavathi Viswanadhapalli, Uday P. Pratap, Ratna K. Vadlamudi

**Affiliations:** 1 Department of Obstetrics and Gynecology, University of Texas Health San Antonio, San Antonio, TX 78229, USA; 2 Mays Cancer Center, University of Texas Health San Antonio, San Antonio, TX 78229, USA; 3 Audie L. Murphy Division, South Texas Veterans Health Care System, San Antonio, TX 78229, USA

**Keywords:** ferroptosis, breast neoplasms, glutathione peroxidase 4, amino acid transport systems, iron metabolism disorders, lipid peroxidation, tumor microenvironment, triple negative breast neoplasms, drug resistance, neoplasm

## Abstract

Ferroptosis is a regulated form of cell death driven by iron-dependent lipid peroxidation and insufficient antioxidant defenses and is mechanistically distinct from apoptosis, necroptosis, and other cell death mechanisms. Over the past decade, ferroptosis has emerged as a significant determinant of cancer cell fate. An increasing body of research indicates that it may serve as a vulnerability in breast cancer treatment. Breast cancers undergo significant metabolic and redox reprogramming that directly influences ferroptosis regulation, including alterations in iron homeostasis, polyunsaturated lipid metabolism, and antioxidant networks. Sensitivity to ferroptosis varies among breast cancer subtypes, underscoring subtype-specific metabolic requirements and stress-response mechanisms. Ferroptosis plays a critical role in breast cancer stem cells (BCSCs), therapeutic resistance, and tumor recurrence. Targeting ferroptosis provides a promising therapeutic strategy to eradicate drug-resistant, stem-like populations. Ferroptosis also profoundly influences the tumor microenvironment (TME) by altering immune cell function, reshaping stromal cell interactions, and modulating cellular responses to hypoxia and metabolic stress. This review summarizes the current mechanistic insights into ferroptosis regulation in breast cancer and discusses therapeutic avenues targeting breast cancer cells, stem cells, and the tumor microenvironment. Understanding ferroptosis mechanisms in breast cancer subtypes may enable rational, biomarker-guided strategies to overcome therapeutic resistance and improve clinical outcomes for patients with breast cancer.

## 1. Introduction

Breast cancer is the most diagnosed malignancy in women worldwide and is responsible for about 685,000 deaths annually, accounting for approximately 6.9% of all cancer-related mortality [1]. Breast cancer comprises distinct molecular subtypes, including estrogen receptorpositive (ER^+^), HER2-positive (HER2^+^), and triple-negative breast cancer (TNBC), each characterized by specific genetic, epigenetic, and metabolic features that shape prognosis and therapeutic response [2–4]. Approximately 60–70% of breast cancer patients are ER^+^, 10–15% are HER2^+^, and 15–20% are TNBC [5]. Despite advances in early detection, endocrine therapy, HER2-targeted agents, and immunotherapy, durable clinical benefit is frequently undermined by tumor heterogeneity, metabolic plasticity, and the emergence of therapy-resistant disease [6,7]. Therefore, there is an urgent need to develop new therapeutic strategies for breast cancer.

Ferroptosis is a regulated, non-apoptotic form of cell death that occurs when iron-dependent lipid peroxidation overwhelms cellular antioxidant defenses, particularly the cystine-glutamate antiporter (system x_c_^*−*^)–glutathione (GSH)–glutathione peroxidase 4 (GPX4) axis [8]. This mechanism is controlled by a network of antioxidant systems, ironhandling proteins, and stress-responsive signaling pathways [9]. It is strongly associated with cellular metabolism, redox balance, and membrane lipid composition, in contrast to classical apoptotic pathways, which are frequently altered in breast cancer [10–12]. Growing evidence indicates that ferroptosis is a key vulnerability across breast cancer subtypes including ER^+^, HER2^+^, TNBC with subtype-specific differences in sensitivity driven by distinct metabolic and antioxidant programs [11,13]. Ferroptosis also regulates breast cancer stem cells (BCSCs), drug resistance, and interactions within the tumor microenvironment (TME), such as those between immune and stromal cells [12,14]. These characteristics implicate ferroptosis at the convergence of tumor cell-intrinsic susceptibilities and extrinsic microenvironmental stresses.

Oxidative stress, defined as a relative excess of reactive oxygen species (ROS) relative to antioxidant defenses, is implicated in numerous pathological conditions [15]. Obesity, a major risk factor for metabolic diseases such as diabetes, cardiovascular disorders, and breast cancer, is strongly associated with elevated oxidative stress [16,17]. Moreover, oxidative stress is a hallmark of cancer, contributing to genomic instability, oncogenic signaling, and metabolic reprogramming that drive tumor initiation and progression [18]. Excess ROS promote irondependent lipid peroxidation of phospholipid membranes and the accumulation of lipid hydroperoxides, particularly in polyunsaturated phospholipids, forming the biochemical basis of ferroptosis, a regulated cell death pathway distinct from apoptosis and necroptosis [19,20]. Cancer cells, which often endure chronic oxidative stress, are particularly vulnerable to therapies that further disrupt redox homeostasis and GSH/GPX4-dependent detoxification, shifting ROS from pro-tumorigenic signaling toward lethal ferroptotic cell death [21,22]. In summary, oxidative stress plays a central role in cancer progression, and therapeutic strategies that exploit redox imbalance can effectively induce ferroptosis in cancer cells.

The purpose of this review is to synthesize current mechanistic insights into ferroptosis regulation in breast cancer and to evaluate emerging therapeutic strategies targeting breast cancer cells, stem cell populations, and the tumor microenvironment.

## 2. Ferroptosis: Definition, Key Features, and Implications in Breast Cancer

### 2.1 Overview of Ferroptosis

Ferroptosis is a controlled, non-apoptotic type of cell death that occurs when lipid peroxides build up to harmful levels in iron-dependent cell membranes [8,23] and is defined by catastrophic lipid peroxidation, distinguishing it from apoptosis, necroptosis, and pyroptosis [24]. Ferroptosis is biochemically defined by three characteristics: (i) the presence of redox-active iron, (ii) the availability of polyunsaturated phospholipid substrates, and (iii) the inadequacy of lipid-peroxide detoxification mechanisms, notably GPX4 and its upstream GSH supply [25]. Redox-active iron, sourced from labile iron pools and iron-containing enzymes, catalyzes the generation of ROS and facilitates lipid peroxidation via both Fenton chemistry and lipoxygenase-dependent mechanisms, resulting in oxidative damage to phospholipid bilayers [4]. If antioxidant defenses like GPX4-mediated reduction of phospholipid hydroperoxides fail because GSH is low, GPX4 is directly blocked, or cystine can’t get into the cells, cells undergo ferroptotic death due to lipid ROS accumulation [25].

Morphologically, biochemically, and genetically, ferroptosis was differentiated from apoptosis, necrosis, and autophagy, and represents a distinct form of programmed cell death [23]. Ferroptotic cells exhibit smaller mitochondria, thicker membranes, and missing mitochondrial cristae [8]. Ferroptosis is mechanistically distinct from necrosis, which is characterized by loss of membrane integrity, organelle swelling, and eventual plasma membrane rupture. Iron chelators or lipophilic antioxidants can specifically inhibit ferroptosis, suggesting that it is a metabolically encoded susceptibility rather than merely a result of cellular damage. All of these factors make ferroptosis a promising target for cancer treatment [25].

Increasing evidence positions ferroptosis as a therapeutic vulnerability across multiple breast cancer subtypes including TNBC, ER^+^, HER2^+^, and inflammatory breast cancer (IBC) [11,26,27]. Ferroptosis was identified as a pivotal vulnerability in breast cancer, associated with key elements that contribute to unfavorable clinical outcomes, such as pronounced tumor heterogeneity, metabolic adaptability, and resistance to conventional treatments [28,29]. Different subtypes of breast cancer exhibit differential regulation of key ferroptosis proteins such as GPX4, FSP1, transferrin receptor-1 (TFR1), and ferritin [30]. Moreover, acyl-CoA synthetase long-chain family member 4 (ACSL4), which affects the makeup of polyunsaturated phospholipids is more common in TNBC tumors [31]. Collectively, these emerging findings suggest that ferroptosis may be a promising candidate for targeted therapy and may serve as a molecular basis for understanding breast cancer heterogeneity and drug resistance.

### 2.2 Molecular Mechanisms of Ferroptosis

At the molecular level, ferroptosis is driven by the iron-dependent accumulation of lipid peroxides and is regulated by a network of antioxidant systems, iron-handling proteins, and stressresponsive signaling pathways [9]. The system x_c_^*−*^–GSH–GPX4 axis is pivotal in the inhibition of ferroptosis, wherein Solute Carrier Family 7 Member 11 (SLC7A11) facilitates the import of cystine for GSH production, and the selenoenzyme GPX4 employs GSH to convert phospholipid hydroperoxides into their respective alcohols, thus averting detrimental membrane damage. In breast cancer, high SLC7A11 and GPX4 activity counteracts oxidative stress and contributes to ferroptosis resistance, while pharmacologic or genetic inhibition of these proteins sensitizes tumor cells to ferroptosis-inducing agents such as erastin and (RAS-Selective Lethal 3) RSL3.

**Fig. 1.**
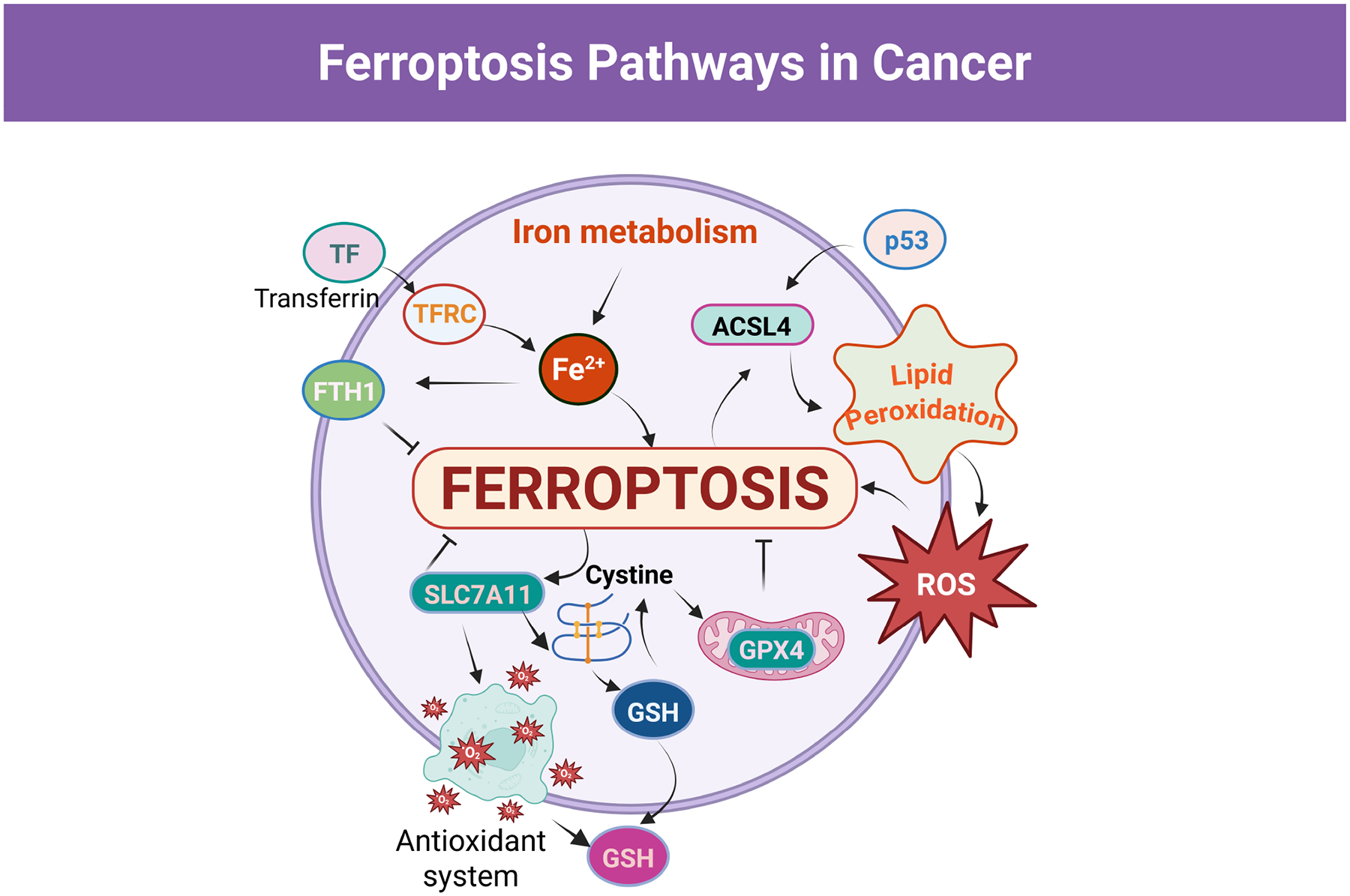
Schematic illustration of the core molecular mechanisms regulating ferroptosis in cancer cells. Created in BioRender.com. Viswanadhapalli, S. (2026) https://BioRender.com/1hfq806. Transferrin (TF) binds to transferrin receptor (TFRC) at the cell surface to mediate iron uptake, increasing intracellular Fe^2+^ and driving mitochondrial and cytosolic reactive oxygen species (ROS) generation, which promotes irondependent lipid peroxidation. Acyl-CoA synthetase long-chain family member 4 (ACSL4) enhances the incorporation of polyunsaturated fatty acids into membrane phospholipids, resulting in the accumulation of lipid hydroperoxides and ferroptotic cell death. Antioxidant defense pathways counteract ferroptosis via SLC7A11-mediated cystine import, glutathione (GSH) synthesis, and glutathione peroxidase 4 (GPX4) dependent detoxification of lipid peroxides. Ferritin heavy chain 1 (FTH1) restricts the labile iron pool and thereby limits ROS production and lipid peroxidation. The tumor suppressor p53 modulates ferroptosis sensitivity by regulating redox homeostasis and lipid metabolic pathways. SLC7A11, Solute Carrier Family 7 Member 11; p53, tumor protein p53.

A parallel, GSH-independent defense is mediated by ferroptosis suppressor protein-1 (FSP1/AIFM2), which regenerates ubiquinol at the plasma membrane to trap lipid radicals; together, GPX4 and FSP1 establish partially redundant antioxidant barriers that must often be cotargeted to achieve robust ferroptosis in cancer cells [9]. By contrast, Acyl-CoA synthetase long-chain family member 4 (ACSL4) promotes ferroptosis by activating polyunsaturated fatty acids (PUFAs) such as arachidonic and adrenic acid for incorporation into phospholipids, creating PUFA-rich membranes that serve as substrates for iron-dependent lipid peroxidation in cooperation with Lysophosphatidylcholine Acyltransferase 3 (LPCAT3) and lipoxygenases [32] (Fig. 1).

Iron metabolism provides the redox-active iron that fuels ROS generation and propagates lipid peroxidation during ferroptosis. Breast cancer cells frequently show up-regulated TFR1 expression and enhanced transferrin-bound iron uptake, coupled with reduced ferroportin-mediated iron export and elevated ferritin levels, thereby expanding the intracellular labile iron pool while buffering overt toxicity [33]. Under ferroptosis-inducing conditions, ferritin may undergo selective autophagic degradation (ferritinophagy), releasing stored iron and further amplifying ROS production via Fenton reactions and iron-containing enzymes [33].

Autophagy, tumor protein p53 (p53) signaling, and endoplasmic reticulum (ER) stress form an additional regulatory layer that integrates metabolic and genotoxic cues to modulate ferroptosis [34,35]. Autophagic processes such as ferritinophagy and lipophagy can promote ferroptosis by mobilizing iron from ferritin and remodeling lipid stores toward PUFA-rich phospholipids, whereas excessive mitophagy or general autophagy in some settings may alleviate oxidative stress and delay cell death [36]. Under conditions of mild lipid peroxidation, p53 may suppress ferroptosis and favor survival or alternative death pathways [37].

ER stress and the unfolded protein response (UPR) intersect with ferroptosis through PERK–eIF2*α*–ATF4–CHOP signaling and related branches, which influence antioxidant genes, system x_c_^*−*^ activity and lipid-metabolic enzymes [38]. In preclinical models, ferroptosis inducers such as erastin or artesunate elicit ER stress and upregulate the pro-apoptotic factor PUMA via CHOP, thereby synergizing with Tumor necrosis factor (TNF)-related apoptosis-inducing ligand (TRAIL) and demonstrating that ER-stress–driven CHOP/PUMA signaling links ferroptosis to apoptotic machinery in a largely p53-independent fashion [39]. Finally, extensive cross-talk connects ferroptosis to other forms of regulated cell death and to immune regulation, with important implications for breast cancer therapy. Shared upstream mediators, including ROS, p53, NF-*κ*B and nuclear factor erythroid 2–related factor 2 (NRF2), coordinate the balance between apoptosis, necroptosis and ferroptosis, while NRF2 in particular upregulates multiple anti-ferroptotic effectors such as SLC7A11, GPX4 and HO-1, thereby contributing to drug resistance [40].

### 2.3 Ferroptosis and Breast Cancer Subtypes

Ferroptosis susceptibility and the translational potential of ferroptosis-targeted therapies appear to vary across breast cancer subtypes. Ferroptosis sensitivity is shaped by subtype-specific metabolic and epigenetic programs ranging from ER-driven antioxidant defenses in luminal tumors, to cysteinedependent redox vulnerabilities in HER2^+^ disease and the heightened yet TME-modulated ferroptosis susceptibility of TNBC. Together, these patterns highlight ferroptosis as a shared but subtype-specific therapeutic avenue across breast cancer.

Luminal A and Luminal B tumors (predominantly ER^+^) rely heavily on antioxidant defenses. Gene-expression studies have identified ferroptosis-related signatures that are differentially expressed in Luminal A disease and associate with prognosis [41]. Compared with more aggressive subtypes, Luminal A cancers often experience lower oxidative stress; nevertheless, ferroptosis remains a key regulatory process in these tumors. SLC7A11 protects cancer cells from ferroptosis and intersects with endocrine responsiveness and tumor–immune interactions [42,43]. Mechanistically, ER signaling directly contributes to ferroptosis resistance. Estrogen/ER*α* elevates system x_c_^*−*^ (SLC7A11/SLC3A2), increasing cystine import and GSH synthesis to suppress lipid peroxidation [42]. Consistent with this biology, ER^+^ tumors are relatively less ferroptosis-prone [44]. In sum, although ER^+^ cancers are comparatively resistant, they harbor actionable ferroptosis vulnerabilities, particularly in the context of endocrine and Cyclin-Dependent Kinases 4 and 6 (CDK4/6)-based therapies.

HER2^+^ tumors also display ferroptosis-linked features. Using TCGA and METABRIC cohorts, Shi *et al*. [45] derived a four-gene ferroptosis-related signature (*SLC7A11*, Prominin 2 (*PROM2*), Fanconi Anemia Complementation Group D2 (*FANCD2*), Familial Hypercholesterolemia (*FH*)). Elevated expressions of *PROM2*, *SLC7A11*, *FANCD2*, and *FH* contribute to a higher ferroptosis-related risk score and are associated with poorer overall survival in HER2^+^ breast cancer, whereas patients with lower expression of this gene signature exhibit significantly improved clinical outcomes. Single-cell RNA-seq localized *SLC7A11*, *PROM2*, and *FANCD2* primarily to malignant epithelial cells, and the high-risk group exhibited lower immune scores, higher tumor mutation burden (TMB), and distinct immune infiltration and drugsensitivity profiles features further refined by combining the ferroptosis score with TMB [45]. Functionally, cysteine metabolism emerges as a therapeutic vulnerability in trastuzumab-resistant HER2^+^ disease. Epigenetic remodeling (increased H3K4me3 and DNA hypomethylation) upregulates *SLC7A11* and cystine uptake, reinforcing antioxidant capacity and ferroptosis resistance [46]. Ferroptosis-related gene signatures carry prognostic value in HER2^+^ breast cancer [45].

Emerging data suggest ferroptosis as both an intrinsic route for tumor cell death and a regulator of antitumor immunity in TNBC. Ferroptosis can enhance immune attack by reshaping tumor-infiltrating immune cells, but inappropriate ferroptosis in certain immune subsets may conversely promote immunosuppression, highlighting the need for context-specific therapeutic strategies. Multi-omics analyses reveal subtype-specific ferroptosis sensitivity: for example, the luminal androgen receptor (LAR) TNBC subtype exhibits increased GSH metabolism and GPX4 activity, rendering it particularly sensitive to GPX4 inhibition [47]. The TME critically shapes ferroptosis sensitivity in TNBC. Cancer-associated fibroblasts (CAFs) suppress ferroptotic death and foster chemoresistance through metabolic–epigenetic coupling: CAF-derived lactate drives histone lactylation (notably H3K18), upregulating *ZFP64*, which then activates *GCH1* and *FTH1* transcription. This pathway limits lipid peroxidation and consumes Fe^2+^, conferring resistance to doxorubicin [48]. Ferroptosis-regulatory genes *GPX4*, *ACSL4*, and *BCAT2* are expressed at lower levels in TNBC tumors compared with normal breast tissue, and their expression levels are positively correlated, suggesting coordinated regulation of ferroptosis-associated metabolic pathways. Clinically, reduced BCAT2 expression correlates with larger tumor size and higher Ki67 proliferation index, indicating that low BCAT2 levels are associated with increased tumor aggressiveness and poorer clinical outcomes, supporting its potential value as a diagnostic and prognostic biomarker [49].

### 2.4 Ferroptosis as a Therapeutic Vulnerability in BCSCs

**Fig. 2.**
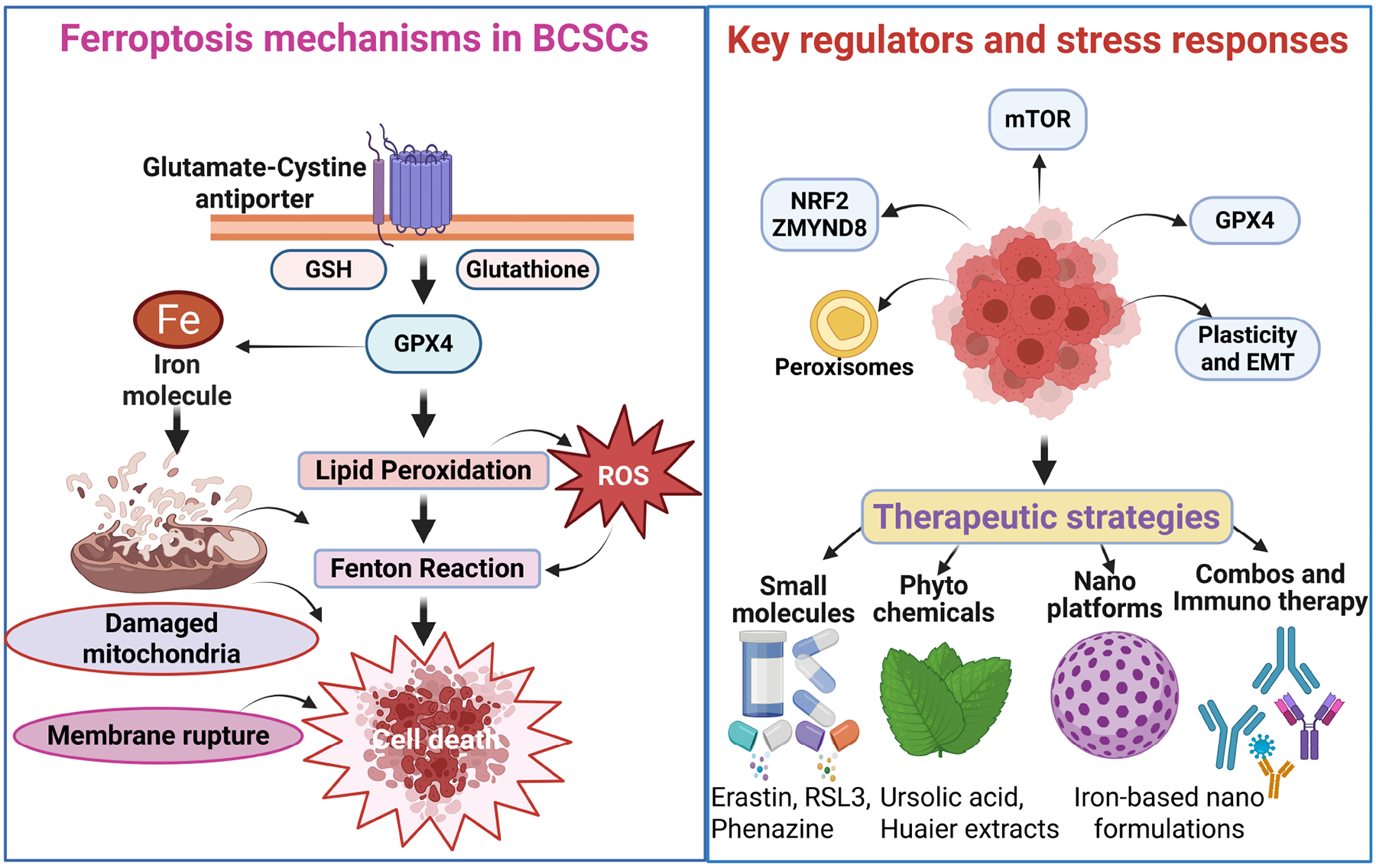
Ferroptosis regulatory networks and therapeutic vulnerabilities in breast cancer stem cells (BCSCs). Created in BioRender.com. Viswanadhapalli, S. (2026) https://BioRender.com/izcd1zp. Left panel: Core ferroptosis machinery in BCSCs, highlighting the system x_c_^*−*^–GSH–GPX4 axis, intracellular iron (Fe^2+^), lipid peroxidation, mitochondrial damage, and ROS accumulation culminating in ferroptotic cell death. Right upper panel: Principal regulatory nodes, including nuclear factor erythroid 2–related factor 2 (NRF2)/zinc finger MYND-type containing 8 (ZMYND8), mechanistic target of rapamycin (mTOR) signaling, peroxisomes, GPX4, and pathways governing cellular plasticity and epithelial–mesenchymal transition (EMT). Right lower panel: Therapeutic strategies targeting these vulnerabilities, encompassing small-molecule ferroptosis modulators, phytochemicals such as ursolic acid and Huaier extract, iron-based nanoplatforms, and combination regimens with immunotherapy. RSL3, RAS-selective lethal 3.

BCSCs represent a highly tumorigenic and therapy-resistant cell population responsible for intratumoral heterogeneity, therapeutic failure, disease recurrence, and metastatic dissemination [50]. Characterized by robust self-renewal capacity and metabolic plasticity, BCSCs survive chemotherapy and radiotherapy through heightened stress-response pathways and adaptive redox metabolism [51]. Emerging evidence indicates that these same metabolic features create a unique dependency on lipid and redox homeostasis, rendering BCSCs selectively vulnerable to ferroptosis, a regulated iron-dependent mode of cell death driven by overwhelming lipid peroxidation [10]. Multiple preclinical studies using diverse breast cancer models, particularly TNBC, highlight ferroptosis induction as a promising approach to eradicate BCSCs and overcome therapeutic resistance [52–57] (Fig. 2).

Central to ferroptotic regulation in BCSCs is the system x_c_^*−*^ (SLC7A11)–GSH–GPX4 axis. BCSCs rely heavily on GPX4-mediated detoxification of phospholipid hydroperoxides, using GSH as an essential reducing equivalent. Inhibition of cystine import via x_c_^*−*^ disrupts intracellular GSH synthesis, leading to unchecked lipid peroxide accumulation and ferroptotic death. Genetic or pharmacologic suppression of system x_c_^*−*^ effectively induces ferroptosis in aldehyde dehydrogenase 1A1 positive (ALDH1A1^+^) BCSCs [52]. Ferroptosis sensitivity in BCSCs is closely linked to epithelial–mesenchymal plasticity. Zinc finger E-box binding homeobox 1 (ZEB1)-driven transcriptional programs suppress GPX4 expression and remodel lipid metabolism toward a ferroptosis-prone state. Loss of the mitochondrial genome encoded mitomiR-3 further exacerbates lipid peroxidation and heightens ferroptosis susceptibility [53]. Conversely, resistance to forkhead box M1 (FOXM1)-targeted therapies correlates with impaired ferroptotic responses, underscoring GPX4 as a critical checkpoint for drug tolerance in stem-like breast cancer cells [54].

Iron metabolism adds an additional layer of regulation over ferroptosis vulnerability in BCSCs. Ferroptosis requires redox-active ferrous iron to catalyze lipid peroxidation; thus, BCSCs must balance iron availability to maintain stemness while preventing oxidative damage. Ferritinophagy and lysosomal iron release expand the labile iron pool, priming cells for ferroptotic death. Drug-resistant BCSCs treated with polyherbal–gold nanoparticle conjugates undergo ferritin degradation, iron accumulation, and ferroptosis induction [55]. Similarly, iron oxyhydroxide (FeOOH)-based nanoplatforms sensitize BCSCs through coordinated GSH depletion, ROS amplification, and iron release [56]. Lysosomal function is tightly integrated with iron homeostasis in BCSCs. The lysosomal Ca^2+^ channel TRPML1 supports BCSC maintenance via lysosome–mitochondria crosstalk. Inhibition of TRPML1 disrupts this axis, elevates iron-dependent oxidative stress, and selectively drives ferroptotic elimination of BCSCs [57].

BCSCs deploy potent transcriptional stress-response networks to counteract ferroptotic pressure, with NRF2 emerging as the dominant regulator of antioxidant and detoxification pathways. NRF2 orchestrates the expression of genes governing cystine uptake, GSH synthesis, lipid metabolism, and iron sequestration. Activation of NRF2 protects BCSCs from ferroptosis, whereas targeting NRF2-linked pathways such as with ursolic acid suppresses BCSCs proliferation through ferroptosis mechanisms [58]. Hypoxia-responsive signaling further reinforces ferroptosis resistance. Hypoxia-induced cystathionine *β*-synthase (CBS) enhances stemness traits while attenuating ferroptosis responses by rewiring sulfur and redox metabolism; inhibition of CBS restores ferroptosis sensitivity and suppresses BCSCs features [17]. Additionally, the chromatin-associated factor ZMYND8 maintains NRF2 activity and shields BCSCs from oxidative injury, thereby conferring ferroptosis resistance [59].

Because ferroptosis is driven by peroxidation of polyunsaturated phospholipids (PUFA-PLs), membrane lipid composition is a major determinant of ferroptosis sensitivity. BCSCs exhibit distinct lipid remodeling programs that modulate ferroptosis thresholds. Pharmacologic agents such as phenazine derivatives reduce stemness by promoting lipid peroxidation [60], and ursolic acid suppresses TNBC BCSCs proliferation through NRF2-dependent ferroptosis pathways [58]. Peroxisomes have also emerged as key modulators of ferroptosis in BCSCs. Peroxisome-derived ether lipids, along with ACSL and arachidonic acid lipoxygenases (ALOX) family enzymes, shape the lipid peroxide landscape in a context-specific manner [61]. Multifunctional nanoplatforms capable of simultaneously altering cholesterol metabolism and inducing ferroptosis show strong potential for overcoming drug resistance in CSC-like populations [62].

Multiple oncogenic and stress-responsive pathways interface with ferroptosis to regulate BCSCs maintenance. Among these, Hippo/YAP signaling plays a critical role: YAP enhances stemness and drug resistance while regulating redox homeostasis and lipid metabolic genes. Consequently, targeting YAP may potentiate ferroptosis-based therapeutic strategies [63]. Caveolin-1 (CAV1) also contributes to this regulation; it suppresses xCT in an IFNGR1dependent manner, thereby promoting ferroptosis, reducing stemness, and improving responsiveness to anti-programmed cell death protein-1 (anti-PD-1) therapy [64]. mTOR signaling further modulates ferroptosis in BCSCs. Although mTOR supports BCSCs survival, its inhibition can paradoxically attenuate salinomycin-induced ferroptosis by stabilizing mitochondrial function, underscoring the importance of treatment context and scheduling [65,66].

In summary, BCSCs exhibit a pronounced dependence on redox balance, lipid metabolism, and iron homeostasis, rendering them uniquely susceptible to ferroptosis despite their broad resistance to conventional therapies. Disruption of core antioxidant defenses, ironhandling pathways, and lipid-peroxidation control mechanisms effectively induces ferroptotic death in BCSCs, highlighting ferroptosis targeting as a promising strategy to eliminate stemlike, therapy-resistant tumor populations and prevent recurrence.

### 2.5 Ferroptosis and TME

Ferroptosis susceptibility in breast cancer is not governed solely by intrinsic tumor factors. TME elements including immune cells, fibroblasts, adipocytes, hypoxia, and metabolic reprogramming critically shape ferroptosis fate [11,30,67]. These interactions introduce both therapeutic opportunities and vulnerabilities within the breast TME.

#### 2.5.1 Immune Modulation by Ferroptosis in Breast Cancer

Beyond its role as a cell death pathway, ferroptosis functions as a key immunomodulatory process in the TME. Ferroptotic cancer cells can shape both pro-tumoral and anti-tumoral immune responses, underscoring a context-dependent duality [68,69]. Induction of ferroptosis has been shown to suppress breast cancer progression, bypass chemoresistance, and enhance the efficacy of immune checkpoint inhibitors [70]. This dual nature reflects a complex interplay: ferroptosis can stimulate anti-tumor immunity by promoting immune activation, however, excessive lipid peroxidation and iron-driven oxidative stress may also induce immunosuppression and chemoresistance [71]. Ferroptotic cancer cells release damage-associated molecular patterns (DAMPs) which enhance immune recognition and tumor clearance [71]. Clinically, this duality becomes evident when comparing neoadjuvant versus metastatic settings. During neoadjuvant therapy, ferroptosis may enhance tumor shrinkage and immune activation, improving therapeutic responses. Conversely, in metastatic relapse, ferroptosis-associated immunosuppression may promote resistance and therapeutic failure [72]. Thus, ferroptosis simultaneously drives pro- and anti-tumor immune activities depending on the metabolic and microenvironmental context.

#### 2.5.2 Ferroptosis in Immune Cells and Its Impact on Anti-tumor Immunity

Ferroptosis also directly influences immune cells within the TME. Immune effector cells depend on tightly regulated iron flux and lipid metabolism, rendering them sensitive to ferroptotic stress under oxidative or metabolic pressure [73]. Depending on the cell type and metabolic state, ferroptosis may either strengthen anti-tumor immune responses or impair immune function [74]. Different immune cell subsets exhibit varying degrees of ferroptosis sensitivity based on membrane lipid composition. T cells, particularly intratumoral cluster of differentiation 8 positive (CD8^+^) T cells, are highly vulnerable due to their elevated polyunsaturated phospholipid content [75,76]. Although CD8^+^ T cells secrete interferon-gamma (IFNg) to sensitize cancer cells to ferroptosis, they paradoxically undergo ferroptosis more readily than tumor cells [75]. Overall, these findings highlight the need for ferroptosis-targeted approaches that selectively induce lipid peroxidation in tumor cells while preserving immune cell fitness [77].

#### 2.5.3 Hypoxia and Metabolic Stress in the TME

**Fig. 3.**
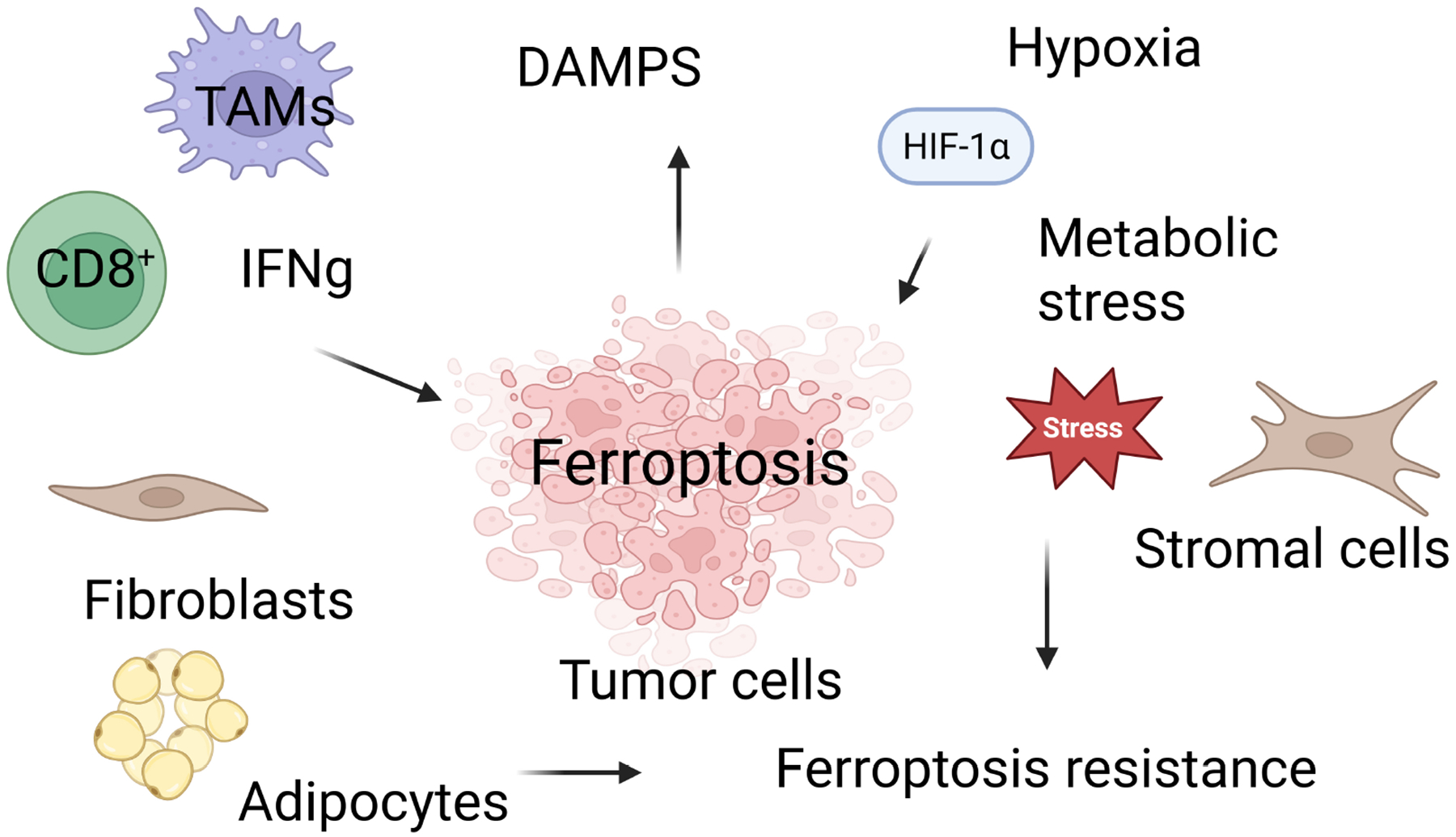
Ferroptosis and key tumor microenvironmental determinants shaping ferroptotic sensitivity in breast cancer. Created in BioRender.com. Vadlamudi, R. (2026) https://BioRender.com/0tk1ik2. This schematic illustrates how diverse components of the breast tumor microenvironment (TME) include immune cells, stromal fibroblasts, adipocytes, hypoxia, and metabolic stress collectively regulate ferroptosis susceptibility. CD8^+^ T cells promote ferroptosis in tumor cells through the secretion of IFNg, yet they are themselves vulnerable to lipid peroxidation-driven ferroptotic death within the oxidative TME. Stromal cells and metabolic interactions, including fibroblasts and adipocyte-derived lipids, modulate tumor ferroptotic fate by providing metabolic substrates and engaging protective antioxidant pathways. Hypoxia-induced HIF-1*α* signaling and metabolic stress pathways including altered lipid metabolism, glutathione/cysteine remodeling, and mitochondrial adaptation drive ferroptosis resistance and facilitate tumor progression. Collectively, these microenvironmental factors determine whether ferroptosis acts as a pro-tumoral or anti-tumoral mechanism within breast cancer. TAMs, tumor-associated macrophages; DAMPs, damage-associated molecular patterns; IFNg, Interferon-gamma; HIF-1*α*, hypoxiainducible factor-1 alpha.

Hypoxia, a defining characteristic of the TME, is a major driver of ferroptosis resistance in breast cancer. Hypoxia-inducible factor-1 (HIF-1) reshapes cellular metabolism to reduce susceptibility to ferroptotic death [78]. TNBC cells, for example, gain ferroptosis resistance through HIF-1–mediated induction of CBS, which alters cysteine–glutathione metabolism and protects against cystine-deprivation-induced ferroptosis [79]. Other studies likewise indicate that hypoxia broadly suppresses ferroptosis in breast cancer [80], although ferroptosis induction can enhance radiosensitivity in hypoxic tumors by amplifying oxidative stress [81]. Metabolic stress further reinforces ferroptosis resistance through mitochondrial remodeling. As the primary site of iron utilization and ROS generation, mitochondria critically regulate ferroptotic sensitivity [30,82]. Mitochondria-targeted ferroptosis inducers such as IR780-SPhF demonstrate potent preclinical antitumor activity and reveal new opportunities for therapeutic intervention [83]. These adaptations are particularly pronounced in TNBC [84]. Collectively, hypoxia and metabolic stress create ferroptosis-resistant niches within breast tumors, especially aggressive TNBC, highlighting the dominant influence of the TME over intrinsic tumor ferroptotic vulnerability [85]. These factors must be accounted for when designing ferroptosis-based therapeutic strategies (Fig. 3).

### 2.6 Therapeutic Strategies Targeting Ferroptosis

**Fig. 4.**
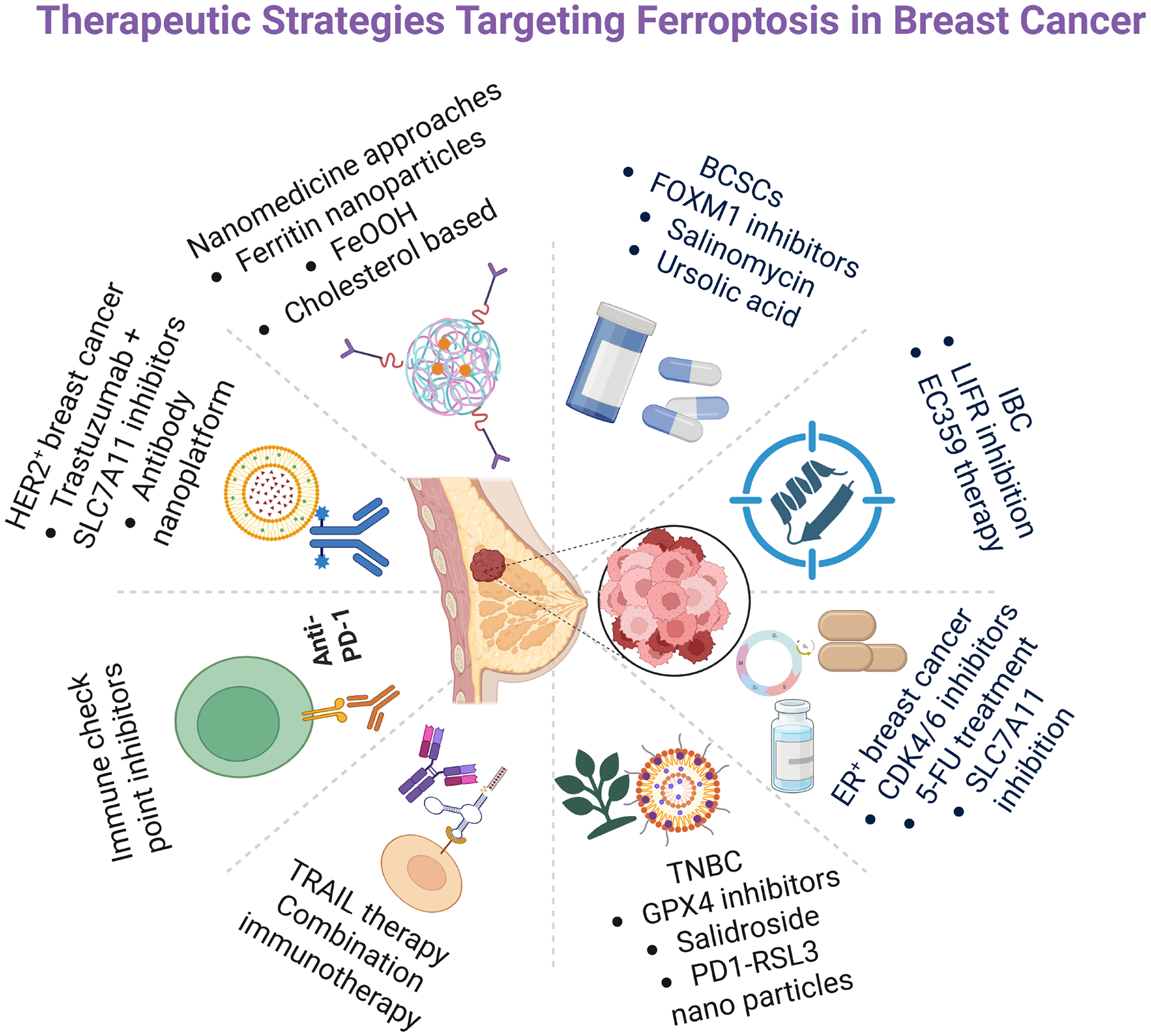
Therapeutic strategies targeting ferroptosis in breast cancer. Created in BioRender.com. Viswanadhapalli, S. (2026) https://BioRender.com/izcd1zp. A schematic overview summarizing major therapeutic approaches designed to induce ferroptosis across breast cancer subtypes. Strategies include targeting SLC7A11, GPX4, and cystine metabolism in estrogen receptor–positive (ER^+^) and human epidermal growth factor receptor-2 positive (HER2^+^) tumors; GPX4 inhibition, salidroside, tumor necrosis factor (TNF)-related apoptosis-inducing ligand (TRAIL)-based combinations, and programmed cell death protein-1 (PD-1)/RSL3 nanoparticle approaches in triple-negative breast cancer (TNBC); LIFR inhibition and EC359 therapy in inflammatory breast cancer (IBC); and BCSC-directed interventions such as FOXM1 inhibitors, salinomycin, and ursolic acid. Nanomedicine-based tactics including ferritin nanoparticles, iron oxyhydroxide (FeOOH) systems, and cholesterol-modulating platforms further enhance ferroptosis induction. Collectively, these modalities exploit redox imbalance, iron-handling pathways, and lipid-peroxidation mechanisms to overcome therapeutic resistance in breast cancer. FOXM1, forkhead box M1; LIFR, leukemia inhibitory factor receptor; EC359, small molecule inhibitor of leukemia inhibitory factor receptor; CDK4/6, Cyclin-Dependent Kinases 4 and 6; 5-FU, 5-fluorouracil.

Ferroptosis has emerged as a promising therapeutic axis in oncology [86]. In breast cancer, ferroptosis induction has been shown to inhibit proliferation and invasion, overcome resistance to chemotherapy and targeted agents, and potentially enhance tumor immunogenicity via the release of damage-associated molecular patterns that engage immune cells [40]. Combination strategies that co-target ferroptosis and immune evasion, such as pairing ferroptosis inducers with immune checkpoint inhibitors, or TRAIL are increasingly attractive for reshaping the TME and improving responses in aggressive subtypes [9]. Consistent with a role for lipid metabolic wiring, tumors and cell lines with elevated ACSL4 expression display heightened ferroptosis sensitivity [12] (Fig. 4).

#### 2.6.1 Targeting Ferroptosis in ER^+^ Breast Cancer

In ER^+^ breast cancer, therapeutic stress can tip cells toward ferroptosis. CDK4/6 inhibitors central to ER^+^ treatment reduce SLC7A11 activity, deplete GSH, and increase lipid peroxidation, thereby inducing ferroptosis [43]. Similarly, 5-fluorouracil promotes ferroptosis through effects on SLC7A11 and GPX4, leading to iron accumulation and lipid peroxidation [87]. Tumors often counter these pressures by upregulating antioxidant programs (notably SLC7A11) to maintain redox balance; pharmacologic or genetic inhibition of SLC7A11 reverses this adaptation and heightens ferroptosis sensitivity, implicating ferroptosis as both a contributor to therapy resistance and a compelling co-target for combination therapy regimens. Experimental nanoplatforms that leverage ferritin, ferritinophagy, or iron-releasing carriers increase intratumoral iron, amplify lipid peroxidation, and synergize with chemotherapeutics in breast cancer models highlighting iron handling as both a vulnerability and a translational lever [88]. In ER^+^ breast cancer, ferroptosis based tactics may help overcome endocrine resistance [42].

#### 2.6.2 Targeting Ferroptosis in HER2^+^ Breast Cancer

In HER2^+^ models, inhibiting SLC7A11 or restricting cystine synergizes with trastuzumab, elevating lipid peroxidation and inducing cell death in resistant cell lines (e.g., JIMT1, SKBR3) [32]. Targeting cystine/SLC7A11 can resensitize trastuzumab-resistant disease [46]. In HER2^+^ tumors, ferroptosis induction may counter resistance to targeted therapies [86]. Innovative delivery approaches further support translation. Gao *et al*. [89] engineered an antibody targeted nanoplatform (FEH), that co-delivers trastuzumab, erastin (a system x_c_^*−*^ inhibitor), and Fe^3+^ within an iron–based metal–organic framework; within the TME. Trastuzumab enhances targeting, erastin suppresses GSH synthesis, and Fe^3+^ is reduced to Fe^2+^ to fuel Fenton chemistry boosting ROS, downregulating GPX4, and driving ferroptosis with improved specificity and antitumor efficacy [89].

#### 2.6.3 Targeting Ferroptosis in TNBC

TNBC is generally more ferroptosis sensitive than ER^+^ or HER2^+^ disease [90]; within TNBC, the luminal androgen receptor (LAR) subtype is particularly susceptible due to increased oxidized phosphatidylethanolamines and impaired GSH metabolism, and preclinical studies show that combining GPX4 inhibition with immune checkpoint blockade (ICB) offers a promising avenue for these patients [30,91,92]. Salidroside (Sal) counteracts ferroptosis evasion by simultaneously inhibiting SCD1 mediated lipogenesis (reducing monounsaturated fatty acids that buffer peroxidation) and enhancing nuclear receptor coactivator 4 (NCOA4) dependent ferritinophagy (increasing intracellular Fe^2+^), thereby elevating lipid peroxidation and ROS to sensitize TNBC and overcome CAF mediated chemoresistance [48,93]. GPX4 inhibitors not only trigger tumor cell ferroptosis but can also augment immune checkpoint blockade: PD-1–membrane coated nanoparticles delivering RSL3 (PD-1@RSL3 nanoparticles [NPs]) potentiate PD-L1 blockade by promoting ferroptosis and antitumor immunity, delaying tumor growth and improving survival; tumor specific GPX4 degradation likewise intensifies ferroptosis initiated immune responses and sensitizes tumors to PD-L1 ICB *in vivo* models [94]. Together, these data position ferroptosis induction alone and in combination with ICB, as a compelling strategy for TNBC.

#### 2.6.4 Targeting Ferroptosis in IBC

IBC is a rare and aggressive form of breast cancer that can arise within all three major molecular subtypes, but it is most frequently associated with HER2^+^ and TNBC [95]. Leukemia inhibitory factor receptor (LIFR) inhibition either genetically or with the small molecule inhibitor of LIFR, EC359 suppresses LIF/LIFR signaling, reduces IBC cell growth and invasion, and promotes apoptosis in a manner reversed by the ferroptosis inhibitor Fer 1. Mechanistically, EC359 disrupts glutathione dependent antioxidant defenses via GPX4 downregulation and significantly reduces xenograft tumor growth, highlighting LIFR targeted ferroptosis induction as a promising strategy for IBC [27].

#### 2.6.5 Targeting Ferroptosis in BCSCs

Ferroptosis intersects deeply with BCSCs biology through networks governing redox balance, iron metabolism, lipid remodeling, and stress response transcription. Although BCSCs deploy robust ferroptosis evasion programs, convergent strategies spanning small molecules, natural products, and nanotechnology show promise in dismantling these defenses. Integrating ferroptosis induction with pathway targeted agents and immunotherapy may eradicate stem-like, therapy resistant populations and prevent relapse, provided timing, delivery, and safety are carefully optimized. Natural compounds (e.g., ursolic acid, phenazine derivatives) directly elevate lipid peroxidation and trigger ferroptosis [58,60]; salinomycin induces ferroptosis in BCSCs but is modulated by mTOR, emphasizing treatment sequencing [65]; and resistance to FOXM1 inhibitors correlates with impaired ferroptosis, suggesting that pairing FOXM1 targeted therapy with ferroptosis inducers may restore sensitivity [54].

#### 2.6.6 Targeting Ferroptosis Using Nanomedicine

FeOOH based systems sensitize BCSCs via GSH depletion, ROS amplification, and iron release [56]; cholesterol modulating multifunctional platforms co-induce ferroptosis to overcome drug resistance [62]; and ferritin nanoparticles co-loaded with lapatinib and PAB eradicate ECM detached TNBC clusters by modulating EGFR signaling and enhancing redox lethality [96]. A self-assembling nanoprodrug capable of co-delivering ferroptosis-inducing agents, differentiation inducers, and chemotherapeutics has been reported for the simultaneous targeting of TNBC stem cells and bulk TNBC [97]. Beyond nanotechnology, Huaier polysaccharides induce autophagy dependent ferroptosis in TNBC BCSCs, underscoring the interplay between autophagy, iron mobilization, and lipid peroxidation [98]. Targeting lysosomal TRPML1 channels and hypoxia driven CBS signaling further reinforces ferroptosis based BCSCs elimination [57,79].

## 3. Conclusion and Perspectives

Noteworthy progress has been made in understanding ferroptosis. The therapeutic relevance of ferroptosis varies across breast cancer subtypes. ER^+^ breast tumors exhibit relative ferroptosis resistance due to estrogen-driven antioxidant programs, yet they show exploitable vulnerabilities under therapeutic stress or SLC7A11 inhibition. HER2^+^ cancers display prognostic ferroptosis related gene signatures and targetable metabolic dependencies, particularly involving cystine/SLC7A11. TNBC exhibits ferroptosis heterogeneity; although intrinsically more sensitive, its susceptibility is heavily shaped by the TME, including CAF mediated metabolic rewiring, immune cell ferroptosis sensitivity, and hypoxia driven resistance. The dual immunological effects of ferroptosis complicate the design of effective breast cancer therapies. Future studies are needed to fully understand the interplay between metabolic stress, lipid remodeling, and TME. Future research should also focus on subtype-specific ferroptosis strategies, the development of small molecules that uniquely target ferroptosis drivers present in the subtypes, the identification of clinically actionable biomarkers, and the integration of ferroptosis induction with immunotherapy, targeted therapy, and nanotechnology platforms. Future studies should focus on understanding mechanisms and identifying key players of ferroptosis in breast cancer cells, stem cells, and the TME is essential for developing effective therapies. Mechanistic comprehension of ferroptosis in BCSCs and the TME is essential for formulating effective, individualized treatment protocols.

## Abbreviations

ACSL4: acyl-CoA synthetase long-chain family member 4
AIFM2/FSP1: ferroptosis suppressor protein 1
ALDH1A1: aldehyde dehydrogenase 1 family member A1
BCSCs: breast cancer stem cells
CAF: cancer-associated fibroblast
CAV1: caveolin-1
CBS: cystathionine *β*-synthase
CDK4/6i: CDK4/6 inhibitors
DAMPs: damageassociated molecular patterns
ER^+^: estrogen receptor–positive
ER stress: endoplasmic reticulum stress
Fer-1: ferrostatin-1
FeOOH: iron oxyhydroxide
FPN: ferroportin
GPX4: glutathione peroxidase 4
GSH: glutathione
HER2^+^: human epidermal growth factor receptor-2 positive
HIF-1: hypoxia-inducible factor-1
IBC: inflammatory breast cancer
ICB: immune checkpoint blockade
LAR: luminal androgen receptor
LOX: lipoxygenase
mTOR: mechanistic target of rapamycin
NCOA4: nuclear receptor coactivator 4
NRF2: nuclear factor erythroid 2–related factor 2
NPs: nanoparticles
PD-1/PD-L1: programmed cell death protein-1/ligand-1
PUFA: polyunsaturated fatty acid
PUFA-PL: polyunsaturated phospholipid
ROS: reactive oxygen species
System x_c_^*−*^: cystine–glutamate antiporter
TAM: tumor-associated macrophage
TMB: tumor mutation burden
TME: tumor microenvironment
TNBC: triple-negative breast cancer
TFR1: transferrin receptor-1
UPR: unfolded protein response
ZEB1: zinc finger E-box binding homeobox 1
ZMYND8: zinc finger MYND-type containing 8
